# Injectable 0.19-mg fluocinolone acetonide intravitreal implant for the treatment of non-infectious uveitic macular edema

**DOI:** 10.1186/s12348-019-0168-9

**Published:** 2019-01-29

**Authors:** Lea F. Weber, Stefanie Marx, Gerd U. Auffarth, Alexander F. Scheuerle, Tamer Tandogan, Christian Mayer, Ramin Khoramnia

**Affiliations:** 1 Department of Ophthalmology, University of Heidelberg, INF 400, 69120 Heidelberg, Germany; 2 Department of Ophthalmology, University of Tuebingen, Elfriede-Aulhorn-Straße 7, 72076 Tuebingen, Germany; 3 Department of Ophthalmology, HELIOS Clinic Pforzheim, Kanzlerstraße 2-6, 75175 Pforzheim, Germany

## Abstract

**Background:**

A retrospective observational clinical study to evaluate the safety and effectiveness of the injectable 0.19-mg fluocinolone acetonide intravitreal implant (ILUVIEN) in the treatment of non-infectious uveitic macular edema.

**Results:**

Data are presented from eight patients (11 eyes) with non-infectious uveitic macular edema who were treated with a 0.19-mg fluocinolone acetonide implant. Nine out of 11 eyes were pseudophakic prior to implantation of fluocinolone acetonide implant, and both phakic eyes required cataract surgery during the follow-up period (the median follow-up was 19 months; range, 8–42 months). Effectiveness and safety were assessed from changes in central retinal thickness (measured using spectral domain optical coherence tomography), corrected distance visual acuity, uveitic activity, and intraocular pressure.

The main outcome measures were changes in central retinal thickness, corrected distance visual acuity, uveitic activity, and intraocular pressure. In 11/11 eyes, central retinal thickness improved between months 1 and 3. The mean maximum decrease of central retinal thickness throughout the follow-up period was 168 ± 202 μm (± standard deviation). Nine out of 11 eyes showed an improvement in corrected distance visual acuity (between + 1 and + 8 lines), and 2/11 eyes lost corrected distance visual acuity (− 1 and − 3 lines, respectively). Nine out of 11 eyes presented with inactive inflammation during the follow-up period, and in 1/11 eyes, there was a relapse at month 42. Four out of 11 eyes presented with a relapse of macular edema between months 3 and 8. The mean increase in intraocular pressure was 2.1 ± 4.7 mmHg. Nine eyes were pseudophakic prior to implantation of the injectable fluocinolone acetonide intravitreal implant. Both phakic patients developed a cataract that was treated with cataract surgery in the follow-up period.

**Conclusions:**

In this small case series with long-term follow-up, treatment of non-infectious uveitic macular edema with the injectable fluocinolone acetonide implant was associated with improved central retinal thickness and corrected distance visual acuity and a manageable safety profile. The advantage of this device is the long-term drug release and the fact that it can be injected into the vitreous as a minor surgical procedure, which is in contrast to other treatment options.

## Introduction

Inflammatory cystoid macular edema is a result of the breakdown of the blood-retina barrier and one of the main reasons for loss of vision in patients with non-infectious uveitis [1]. There are a variety of different local and systemic treatments available to treat uveitis, which help to reduce levels of inflammation and to resolve macular edema [2]. Although a broad range of different corticosteroid-sparing treatments is available, used to avoid the well-known steroid side effects, corticosteroids are still an important component in the treatment of uveitis because they are particularly effective in alleviating the inflammation associated with non-infectious uveitis. The wide spectrum of uveitis requires an individualized treatment approach and often combined therapy in some cases, particularly in patients with intolerance to systemic treatments where localized treatments are administered. This is an area where new corticosteroid-based implants have gained growing interest as their sustained effect may help to stabilize the disease activity. The dexamethasone implant (OZURDEX) is a short-acting corticosteroid lasting up to 6 months, and it has been used successfully in the treatment of uveitis over the last few years [3]. The surgically implanted 0.59-mg fluocinolone acetonide implant (RETISERT) is FDA approved but has no EU license. It has also been shown to be effective in the treatment of uveitis [4], but has been associated with higher risks, e.g., raised intraocular pressure and dislocation of the implant [5]. The 0.19-mg fluocinolone acetonide intravitreal implant (FAc implant; ILUVIEN; Alimera Sciences Inc., Alpharetta, USA) is designed to provide a sustained release of 0.2 μg/day of fluocinolone acetonide for up to 3 years. The implant consists of a non-biodegradable polymer with a length of 3.5 mm and a diameter of 0.37 mm. The Fluocinolone Acetonide for Diabetic Macular Edema (FAME) studies showed the FAc implant was effective in patients with diabetic macular edema (DME) and is now licensed for this indication [6]. As this implant has been shown to be effective in DME, it is hypothesized to be effective in treating other inflammatory based conditions such as non-infectious uveitis. The injectable FAc implant has been successfully used in the treatment of patients with non-infectious uveitis in a 2-year follow-up study [7]. Our retrospective observational study therefore aimed to evaluate results from our clinic where it has been used off-label to treat non-infectious uveitic ME since 2013.

## Methods

This study encompasses 11 eyes from 8 patients with non-infectious uveitic ME that were treated with the FAc implant in our department. A retrospective review of patient data was conducted based on clinical examinations obtained between 2013 and 2017 in the Department of Ophthalmology at the University of Heidelberg. The analysis involved the measurements of corrected distance visual acuity (VA), central retinal thickness (CRT) measured using spectral domain optical coherence tomography (SD-OCT; Heidelberg Engineering), intraocular pressure (IOP) measured using Goldmann applanation tonometry, and signs of intraocular inflammation in the anterior and posterior segment of the eye according to the inflammation grading scheme defined by the SUN Working Group [8]. In addition, the follow-up data was analyzed with regard to serious adverse events and safety.

## Results

The median patient follow-up period was 19 months (range 8 to 42 months). Four of the patients (5 eyes) had been diagnosed with uveitis intermedia, three patients (4 eyes) had idiopathic intermediate uveitis, and one patient (one eye) suffered from intermediate uveitis associated with multiple sclerosis. Two patients had posterior uveitis—the first patient was diagnosed with acute zonal occult outer retinopathy (AZOOR; one eye) and the second patient was diagnosed with MCP (Multifocal Choroiditis and Panuveitis) (two eyes). One patient (two eyes) was diagnosed with rheumatoid arthritis-associated panuveitis and one patient (1 eye) was diagnosed with idiopathic vasculitis. Nine eyes were pseudophakic (81.8%), and two eyes (18.2%) were phakic prior to the treatment with the injectable FAc implant. Table 1 summarizes patient demographics, diagnosis, duration of CME, and treatments before the application of the implant. Five patients were female (62.5%), and three were male (37.5%). The median age of the patients was 49 years (range 25 to 65 years). The median duration of chronic macular edema (i.e., receiving regular therapy) before treatment with FAc implant was 36 months (range 18 to 108 months). Prior to the treatment with FAc implant, seven of the patients (ten eyes) had undergone multiple local steroid treatments with periocular or subconjunctival injection of triamcinolone. All eight patients (11 eyes) had been treated with multiple dexamethasone implants before the FAc implant was injected. Five patients were treated with systemic immunosuppressive therapy (cyclosporine, methotrexate, prednisolone, mycophenolate, or combinations) when they received the injectable FAc implant. One of the eyes had undergone vitrectomy and membrane peeling prior to receiving the FAc implant.

**Table 1:** Overview of the diagnosis, duration of CME, and ocular and systemic treatment of the patients prior to FAc implant

No. of eye	Gender, age (years)	Diagnosis	Systemic treatment	Duration of chronic ME under therapy	Previous ocular treatments
1 OD/OS	M, 60	Panuveitis, rheumatoid arthritis	Cyclosporine, methotrexate, prednisolone	36 months	Multiple triamcinolone periocular, multiple dexamethasone intravitreal
2 OS	M, 50	Intermediate uveitis	Mycophenolate	36 months	Multiple triamcinolone periocular and subconjunctival, multiple dexamethasone intravitreal
3 OS	F, 25	Posterior uveitis, acute zonal outer occult retinopathy (AZOOR)	None currently, previously mycophenolate (stopped because of intolerances)	6 years	Multiple dexamethasone intravitreal, bevacizumab (1×) intravitreal
4 OD/OS	F, 48	Intermediate uveitis	None currently, previously methotrexate and mycophenolate, (mycophenolate stopped when diagnosed with malignant melanoma)	4 years	Multiple triamcinolone periocular and intravitreal, multiple dexamethasone intravitreal
5 OD/OS	F, 45	Posterior uveitis, multifocal chorioretinitis and panuveitis (MCP)	Azathioprine	6 years	Multiple triamcinolone periocular and intravitreal, multiple dexamethasone intravitreal
6 OD	M, 50	Intermediate uveitis, multiple sclerosis	Previously: high-dose corticosteroid therapy, interferon beta, fingolimod Currently: rituximab IV every 6 to 9 months	18 months	Triamcinolone periocular (2×), dexamethasone intravitreal (2×)
7 OD	F, 37	Intermediate uveitis	Cyclosporine, methotrexate	9 years	Multiple triamcinolone subconjunctival, multiple dexamethasone intravitreal
8 OD	F, 65	Idiopathic vasculitis	None	25 months	Triamcinolone periocular (1×), multiple dexamethasone intravitreal (3×)

### Central retinal thickness (CRT)

All of the eyes presented with an initial improvement of CRT, and this was observed in 8 eyes in the first 3 months after being treated with the FAc implant. Mean CRT prior to treatment with the FAc implant was 440 μm (range 243 to 930 μm), and the mean maximal decrease in CRT after treatment was 168 μm. Eight eyes (72.7%) presented with a completely dry macula during the follow-up. At the first follow-up point, 1 to 3 months after injection (*n* = 7), there was a mean decrease in CRT of 81 μm (range 10 to 445 μm) and five of the eyes presented with a dry macula. Four to 6 months after the injection (*n* = 6), there was a mean decrease in CRT of 220 μm from baseline levels. Not all of the patients presented for early follow-up examinations in our clinic. One patient was examined for the first time after 6 months, and CRT had decreased by 247 μm with complete resolution of ME (no. 6). Another patient was examined 10 months after injection of FAc implant when CRT had decreased by 31 μm and the macula was completely dry (no. 4, OS). The longest first follow-up time point was 33 months after injection of the FAc implant by which time CRT had decreased and the macula was dry (no. 5, OS). In four cases, CRT increased during the follow-up period. Indeed, one of these patients had a reduction in CRT of 130 μm 1 month after the injection of the FAc implant, but relapsed by month 3 and CRT increased by 81 μm. This was explained partly by the additional presence of an epiretinal membrane (no. 1, OS) with traction in the macular region aside from the uveitic activity. Another case (no. 3) showed a marked initial response to treatment and CRT decreased by 445 μm at month 3 and 698 μm at month 4. There was, however, a relapse of the CRT by month 6 and CRT increased to 610 μm. Nevertheless, there was still a considerable reduction with respect to baseline CRT which was 930 μm. In another case (no. 7), 4 months after the FAc implant was administered, CRT decreased by 168 μm from baseline and there was only minimal intraretinal fluid. By month 8, CRT increased by 118 μm. One of the phakic patients (no. 8) showed a marked reduction in macular edema 6 months after treatment with the FAc implant at which time CRT had decreased by 247 μm and the macula was dry (and then sustained through to month 8). Following cataract surgery, conducted in combination with a pars plana vitrectomy and peeling of the epiretinal membrane, the patient presented with a relapse of ME.

**Fig. 1:**
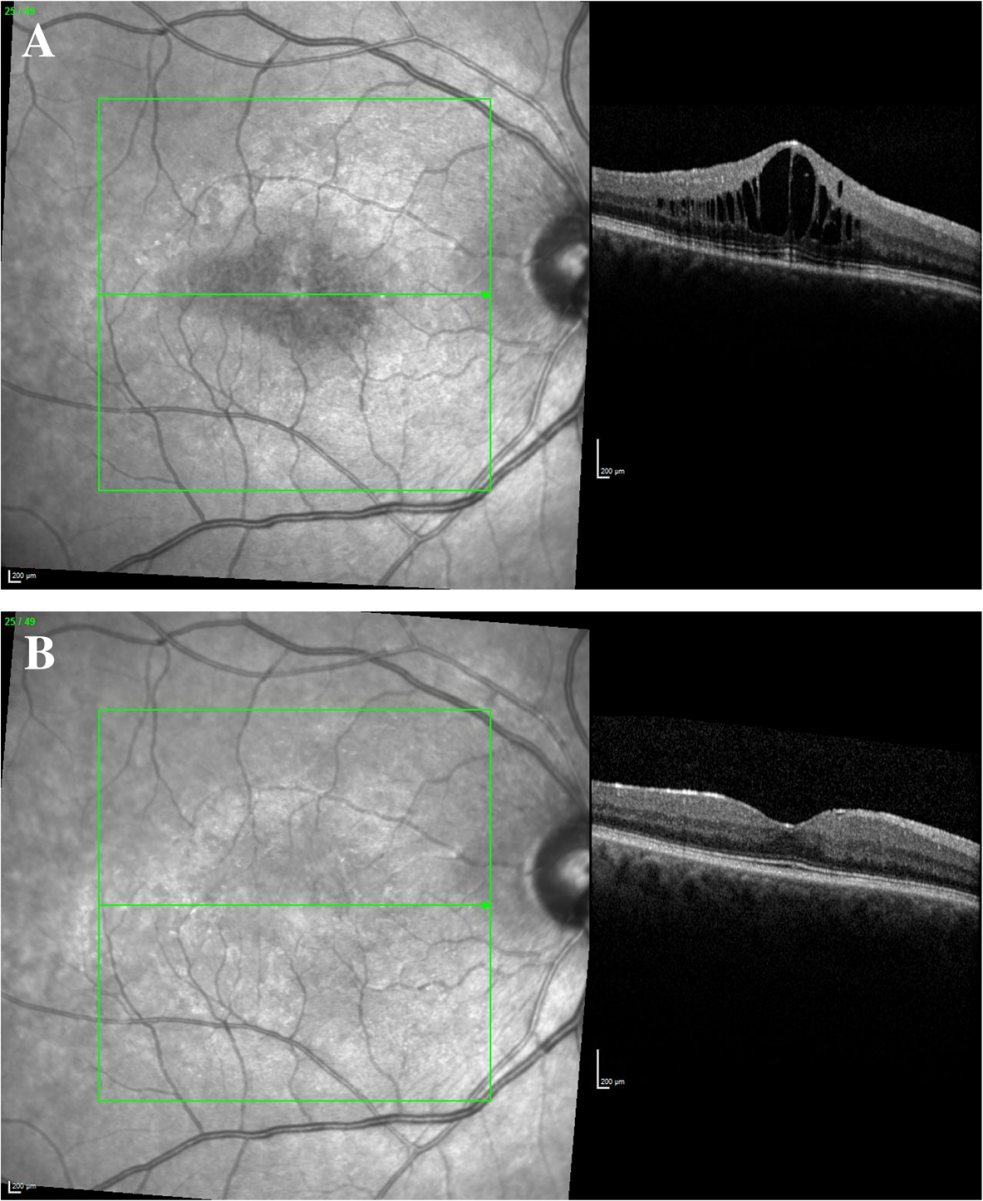
Case no. 1. **a** OD baseline examination: CRT 599 μm, VA 20/40. **b** OD 1-year follow-up: CRT 311 μm, VA 20/25

### Visual acuity (VA)

In our cases, a reduction of CRT was associated with an improvement of VA. Six months after the treatment with the injectable FAc implant, nine of the eyes (81.8%) gained at least one line of VA (1 eye gained 8 lines, 2 eyes gained 3 lines, 2 eyes gained 2 lines, 4 eyes gained 1 line). As our patients suffered from chronic ME prior to treatment with the FAc implant, a full recovery of VA was not observed in all cases. Indeed, a relapse of ME led to a decrease in VA in two patient cases. Also, two phakic eyes experienced a decline in VA during the follow-up period, and in one of these eyes, cataract removal led to an 8 line (from 20/160 to 20/25) improvement in VA.

### Uveitis activity

During the follow-up period, the parameters of uveitis activity (AC-cells, corneal precipitates, vitreous haze, and cells) were recorded and analyzed. Nine of the eyes (81.8%) presented with an improvement or inactive inflammation. One of the eyes (9.1%) failed to respond to treatment and at 3 months presented with macular edema and an increase in vitreous haze (no. 1, OS).

### Duration of the effect

In three patients, the duration of the FAc implant did not last 3 years, and following an initial response, there was a thickening of the macula at months 3, 6, and 8 (no. 1, no. 3, and no. 7, respectively) without a clear cause (e.g., surgery). In a further case, however, relapse of macular edema and vitreous haze occurred 42 months after initial treatment. Responses were maintained in the remaining six eyes (five patients) with stable findings noted at months 16, 19, 32, 33, 35, and 40.

### Adverse events

Both phakic patients developed a cataract after treatment with the FAc implant and required surgery to remove it (Table 2). One of the patients was treated successfully with cataract surgery and went on to experience an improved VA and a dry macula. This patient was treated with prophylactic periocular injection of triamcinolone prior to surgery. The second phakic patient underwent cataract surgery in combination with pars plana vitrectomy and membrane peeling, but developed a macular edema after treatment that required additional therapy. The mean increase in IOP in the whole group was 2.1 ± 4.7 (mean ± standard deviation). None of the patients (*n* = 0) presenting with raised IOP required additional medication or surgery (Table 2). Moreover, no patients presented with major ophthalmic complications (e.g., endophthalmitis, hypotonia, retinal detachment or dislocation of the implant) and no adverse systemic side effects were reported.

**Table 2:** Overview of complications

Complications/surgery	Percentage and number of patients
IOP increase > 5 mmHg	27.3% (*n* = 3/11)
IOP increase > 10 mmHg	9.1% (*n* = 1/11)
IOP increase > 30 mmHg	0% (*n* = 0/11)
IOP lowering surgery	0% (*n* = 0/11)
Cataract surgery	18.2% (*n* = 2/11)

### Individual cases

#### Case no. 1

A male patient aged 60 years with idiopathic non-infectious panuveitis, including significant macular edema in both eyes, received an FAc implant in the right eye and a subsequent FAc implant in the left eye 1 month later. On the day of the insertion, no AC-cells, vitreous haze, or retinal or choroidal lesions were observed. The 1-month follow-up showed a remarkable reduction of CRT from 599 to 320 μm and an improvement of VA from 20/40 to 20/20 in the right eye. The further follow-ups in the right eye showed continuous stable findings in terms of clinical and OCT examinations up until month 19 (Fig. 1). In the left eye, the 1-month follow-up showed a reduction in CRT from 652 to 522 μm, but further follow-ups (3, 4, and 7 months) showed a continuous increase in CRT (up to 642 μm) and signs of inflammation in the vitreous and anterior segment. This was identified by Tyndall, KP, vitreous haze, and vitreous cells which required additional treatment with systemic and local steroids (i.e., subconjunctival TCA at month 7) which led to a reduction of CRT from 642 to 550 μm and an improvement in VA (20/63) as well as remission of intraocular inflammation. Parallel to therapy with the FAc implant, subconjunctival TCA, and systemic steroids, the patient was treated systemically with a stable dose of MTX and CSA. The follow-ups around month 12 showed stable findings in both eyes and so the dose of MTX was reduced from 20 mg/week to 15 mg/week. The different response of both eyes to the FAc implant was explained by the presence of an epiretinal membrane  in the left eye with partial persistence of the edema resulting from traction of the epiretinal membrane.

#### Case no. 2

One 50-year-old female patient with idiopathic non-infectious uveitis intermedia in the left eye was treated with FAc implant. The follow-up (at month 3 and 10) showed a reduction of the CRT from 297 μm (at baseline) to 285 μm at month 10 and that the intraretinal cystoid edema had receded. A reduction of AC cells and vitreous haze was also evident. Anatomical improvements were accompanied by a slight improvement in VA from 20/40 to 20/32. Sixteen months after receiving the FAc implant, the patient presented with stable findings and normal intraocular eye pressure.

#### Case no. 3

One patient had a diagnosis of AZOOR (female, 25 years), and the left eye was treated with the FAc implant. At the 3-month follow-up time point, there was a remarkable reduction of the ME/CRT from 930 to 485 μm and an improvement in VA from 20/400 to 20/160, and intraocular pressure was low/normal. No AC cells or vitreous haze prior or after the treatment were observed. The 4-month follow-up showed a further improvement in ME with a CRT of 232 μm with an increase of the eye pressure to 19 mmHg and VA declining slightly to 20/200. Unfortunately, the 6-month follow-up showed an increase in CRT to 610 μm, which required additional therapy (subconjunctival injection of triamcinolone). At month 8, the CRT decreased to 421 μm, VA was stable, and intraocular pressure was slightly elevated at 22 mmHg.

#### Case no. 4

A 48-year-old female patient suffering from bilateral idiopathic non-infectious uveitis intermedia with diffuse ME and pseudophakia received a FAc implant in both left and right eyes. On the day of insertion, AC cells (1+) and vitreous haze (0.5+) were observed. The right eye was treated first and the 3-month follow-up showed a remarkable improvement in VA from 20/400 to 20/63 and a reduction in CRT from 243 μm to 215 μm, which was accompanied by a reduction of inflammatory signs in the AC and the vitreous. Up to almost 3 years after the injection of FAc implant, the patient presented with stable findings (i.e., a dry macula and low normal eye pressure) and no signs of inflammatory activity in the anterior or posterior segment of the eye. Therapy with mycophenolate (Myfortic) had to be stopped because of the diagnosis of malignant melanoma 4 months after the FAc implant had been injected in the right eye. From month 14, the right eye had no additional local therapy with topical steroids. In the left eye, the FAc implant was injected about 7 months after the right eye had started treatment with the FAc implant. The VA was stable (20/63) after treatment. During follow-up, the cystoid ME receded and the CRT decreased from 280 μm to 249 μm at around month 10 and remained stable up to the last follow-up at month 35. The vitreous cell count decreased (to zero cells), and there were no signs of inflammation in the left eye despite treatment with systemic mycophenolate (Myfortic) being stopped.

#### Case no. 5

A female patient aged 45 years with bilateral multifocal chorioretinitis and panuveitis (MCP) was treated with a single FAc implant in both eyes. On the day of insertion, no AC-cells, vitreous haze, or retinal or choroidal lesions were observed. The right eye, which was treated first, showed a decrease in CRT from 302 to 275 μm at month 1 with stable eye pressure and no signs of inflammatory activity in the anterior or posterior segment of either eye. The patient described the subjective and objective findings during the follow-up as stable and calm. Forty months after the injection of the FAc implant in the right eye and 33 months after it was injected into the left eye, there was a dry macula and no signs of new chorioretinal lesions.

#### Case no. 6

A 50-year-old male patient with uveitis intermedia associated with multiple sclerosis, and cataracta subcapsularis posterior incipiens was treated with a FAc implant (right eye). At month 4, there was a remarkable reduction in CRT from 440 to 247 μm, a dry macula, the vitreous cell count was reduced, there were no signs of inflammatory activity in the anterior or posterior segment of the eye, and there was an improvement of VA from 20/50 to 20/40. The patient presented with stable findings until month 22. Prior to and in parallel to treatment with the FAc implant, rituximab was delivered systemically every 6 to 9 months. The follow-up after 31 months showed a  progression of cataract with a  reduction of the visual acuity (20/160) on the right eye. The patient was treated successfully with cataract surgery and went on to experience an improved VA (20/25) and a dry macula.

#### Case no. 7

A female patient aged 37 years with uveitis intermedia and macula edema was treated with a FAc implant (right eye). On the day of insertion, no AC-cells, vitreous haze, or new chorioretinal lesions were observed. At month 1, CRT decreased from 488 to 407 μm and there was a slight improvement in VA from 20/40 to 20/32. This was accompanied by stable eye pressure and no signs of inflammatory activity in the anterior or posterior segments of the eye. At the 8-month follow-up time point, there was a slight relapse of the macular edema (CRT was 438 μm). Prior to and in parallel to treatment with the FAc implant, MTX was administered systemically.

#### Case no. 8

A female patient with idiopathic non-infectious vasculitis with ME received a single FAc implant in her right eye, which was phakic. On the day of insertion, no AC-cells, vitreous haze, or new chorioretinal lesions were observed. At the 3-month follow-up, there was a decrease in CRT from 536 to 289 μm. No new chorioretinal lesions were reported during the follow-up examinations (up to month 8); however, the right eye did develop a cataract. Because of an additional epiretinal membrane the patient had a cataract surgery in combination with pars plana vitrectomy and membrane peeling. After the surgery, ME developed and required anti-inflammatory therapy in addition to the FAc implant.

## Discussion

Locally delivered steroids have been shown to be effective in the treatment of non-infectious uveitic ME (i.e., the dexamethasone implant). The injectable FAc implant allows a low dose of steroid to be delivered up to 3 years. It can be injected directly into the vitreous unlike other fluocinolone acetonide based implants (e.g., Retisert). In this study, we aimed to determine the effectiveness and safety of the injectable FAc implant in eyes with inflammatory cystoid ME. The results of this case series showed that the intravitreal injection of the FAc implant resulted in a decrease of CRT that was usually accompanied by an improvement in VA and a reduction in uveitis activity. The effect of the treatment was relatively quick with onset occurring between 1 and 3 months. Cases also show that over a relatively long period of time, improvements were sustained (2 eyes > 16 months, 5 eyes > 30 months) and support the long-acting nature of the implant. The extended duration of effect with respect to previously used therapies led to greater patient satisfaction and also a lower rate of consultations with the uveitis specialists. In our examinations, FAc implant presented with an acceptable risk profile and no remarkable increases in IOP were reported. However, both phakic patients developed a cataract requiring surgery during the follow-up period. The FAc implant led to an initial decrease in CRT in all patients. Nevertheless, in some patients, a relapse of ME was reported. In these cases, the dexamethasone implant was successfully used and might indicate that a higher corticosteroid dose may be required in these cases. In one of these patients, a relapse can be explained partly by the additional presence of an epiretinal membrane (no. 1) with traction in the macular region aside from the uveitis activity. Another one of these patients (no. 3) was quite young (25 years) with a high uveitis activity. In these cases, a combination with other treatment options might need to be considered. The FAc implant provided long-term improvement of macular edema and retreatment needs to be considered and planned as they approach 3 years of follow-up. This is particularly important to reduce any potential losses in VA due to reactivation of uveitis.

Local application of intravitreal corticosteroids is an important strategy in the treatment of non-infectious uveitis. This is especially true in unilateral cases of uveitis where local injections are convenient and often lead to fast responses whilst avoiding the systemic side effects of corticosteroids or supplemented immunosuppression. Locally delivered corticosteroids (periocular, subconjunctival or intravitreal injection, or surgical implantation) have shown remarkable results in a number of past studies. Indeed, in a multicenter retrospective cohort study involving 914 patients (1192 eyes), receiving at least one periocular corticosteroid injection (predominantly 40-mg triamcinolone acetonide) reported periocular injections were effective in treating active intraocular inflammation and also reversing declines in VA as a result of DME. Cataract and ocular hypertension occurred in a minority of the cases [9]. As periocular injections only last about 6 weeks, chronic diseases demand longer-lasting options. The Huron study group published their findings in which the dexamethasone intravitreal implant had been used to treat non-infectious intermediate and posterior uveitis [3]. This was a 26-week trial, and eyes with non-infectious intermediate or posterior uveitis and patients were randomized to receive a 0.7-mg dexamethasone implant (*n* = 77), 0.35-mg dexamethasone implant (*n* = 76), or a sham procedure (*n* = 76). Significantly more eyes in the dexamethasone implant-treated arms experienced improved VA than in the sham-treated arm. Moreover, the proportion of eyes with a vitreous haze score of 0 at week 8 was significantly higher in the implant groups than in the sham group. The percentage of eyes with intraocular pressure of ≥ 25 mmHg was 7.1% for the 0.7-mg dexamethasone implant, 8.7% for the 0.35-mg dexamethasone implant, and 4.2% for the sham (*P* > 0.05 at any visit). The incidence of cataract reported in the phakic eyes was 15% (9 of 62 eyes) in the 0.7-mg dexamethasone implant arm, 12% (6 of 51 eyes) in the 0.35-mg dexamethasone implant arm, and 7% (4 of 55) in the sham arm (*P* > 0.05). The authors concluded that a single dexamethasone intravitreal implant significantly improved intraocular inflammation and VA for up to 6 months in patients with non-infectious intermediate or posterior uveitis. The dexamethasone implant has also been successfully used in the treatment of persistent uveitic ME [10].

Longer-acting corticosteroids have been developed such as the surgically placed fluocinolone acetonide implant (Retisert) which was approved by the US FDA in 2005. Its efficacy is being tested in the Multicenter Uveitis Steroid Treatment (MUST) Trial [11]. This study involves 215 patients with intermediate, posterior, or panuveitis that have been treated for 7 years with either a surgically placed intravitreal fluocinolone acetonide implant or systemically administered corticosteroids supplemented with immunosuppression. Follow-up was conducted in tertiary uveitis subspecialty practices. Two hundred fifty-five patients were enrolled in the MUST trial between 2005 and 2008. After 7 years, results showed that the mean change in VA favored systemic therapy (+ 7.2 letters; 95%CI, 2.1–12). A closer look at the data, however, shows that VA in the two groups was not significantly different during the first 5 years. The implant group presented with better results with respect to the effect on uveitis activity and macular edema, but VA results were worse in this group at year 7 due to reactivation of uveitic activity. This seems to be caused by irreversible chorioretinal lesions in the implant group and may possibly relate to severe inflammatory recurrences. It is also worth remembering that the expected duration of the implant was up to 3 years.

So far, the injectable FAc implant is only indicated for the treatment of DME. The FAME trials investigated the safety and efficacy of the FAc implant [12]. This was a multicenter clinical trial involving 375 DME subjects treated with low-dose (0.2 μg/day) and 393 subjects treated with high-dose (0.5 μg/day) fluocinolone acetonide intravitreal implant. Both doses of the FAc implant significantly improved BCVA versus a sham control (*n* = 185) up to 3 years after treatment was initiated [13]. There was a higher rate of cataract surgery in subjects treated with the FAc implants, but interestingly, the VA results after cataract removal were similar to those that were pseudophakic when recruited into the study. Elevated IOP surgery rates were 4.8, 8.1, and 0.5% in low, high, and sham groups. Comparing the relative risk-to-benefit profile, the authors considered the low-dose intravitreal implant to be the better choice as similar efficacy outcomes could be achieved with a lower rate of adverse events.

The injectable FAc implant has also been successfully used in the treatment of patients with non-infectious uveitis in a 2-year follow-up [7]. Data from 11 eyes (11 participants) were randomized to receive either a low- or a high-dose version of a FAc implant. The VA improved significantly by year 2 and none of the study eyes experienced a relapse of the inflammation. Six participants continued to receive systemic medication after implantation and the dosage was reduced in four of them. Five of 11 eyes had received an average of 1.6 periocular triamcinolone acetonide injections in the 12 months preceding implantation. The most common adverse event was elevated IOP. The authors concluded that the implant is a promising approach in patients with non-infectious intermediate and posterior uveitis.

## Limitations

There are several study limitations that need to be mentioned. The data comes from a small, heterogeneous series of patients. There was no randomization and no comparison to a control group (other treatment or sham injection). As the data were analyzed retrospectively, the duration of follow-up was not prespecified. Examinations of the patients were individually planned and executed, in some cases, mainly at the local ophthalmologist’s clinic. This fact, however, can also be seen as a positive effect of the FAc implant that reduced inflammation in these eyes and specialist consultation with a uveitis center was not required. Prior and parallel to the therapy with the FAc implant, five patients (7 eyes) received systemic therapy due to an underlying systemic disease and this may have influenced outcomes as in the majority of cases systemic therapy remained unchanged. Lastly, larger, randomized clinical trials are required to support these findings.

## Conclusions

In this small case series with long-term follow-up, treatment of non-infectious uveitic ME with the injectable FAc implant was associated with improved CRT and VA, as well as reduced uveitis activity and a manageable safety profile but a risk of cataract development. The advantage of this device is the long-term drug release and the fact that it can be injected into the vitreous using a simple surgical injection technique.

## Addendum

The fluocinolone acetonide intravitreal implant is commercially available in Europe, the Middle-East and the USA (ILUVIEN, marketed by Alimera Sciences) for treating DME, and recently received FDA approval in the USA (YUTIQ, marketed by EyePoint Pharmaceuticals) as a treatment for non-infectious uveitis affecting the posterior segment of the eye (NIU-PS).

## Clinical Indications

### ILUVIEN

- a) In Europe, ILUVIEN is indicated for the treatment of vision impairment associated with chronic diabetic macular oedema, considered insufficiently responsive to available therapies;
- b) In the USA and Middle-East, ILUVIEN is indicated for the treatment of diabetic macular edema in patients who have been previously treated with a course of corticosteroids and did not have a clinically significant rise in IOP.

### YUTIQ

Indicated in the USA for the treatment of chronic non-infectious uveitis affecting the posterior segment of the eye.
